# Accurate Neuronal Soma Segmentation Using 3D Multi-Task Learning U-Shaped Fully Convolutional Neural Networks

**DOI:** 10.3389/fnana.2020.592806

**Published:** 2021-01-21

**Authors:** Tianyu Hu, Xiaofeng Xu, Shangbin Chen, Qian Liu

**Affiliations:** ^1^Britton Chance Center for Biomedical Photonics, Wuhan National Laboratory for Optoelectronics, Huazhong University of Science and Technology, Wuhan, China; ^2^MoE Key Laboratory for Biomedical Photonics, School of Engineering Sciences, Huazhong University of Science and Technology, Wuhan, China; ^3^School of Biomedical Engineering, Hainan University, Haikou, China

**Keywords:** touching neuronal soma segmentation, fully convolutional neural network, multi-task learning, micro-optical images, neuronal soma localization

## Abstract

Neuronal soma segmentation is a crucial step for the quantitative analysis of neuronal morphology. Automated neuronal soma segmentation methods have opened up the opportunity to improve the time-consuming manual labeling required during the neuronal soma morphology reconstruction for large-scale images. However, the presence of touching neuronal somata and variable soma shapes in images brings challenges for automated algorithms. This study proposes a neuronal soma segmentation method combining 3D U-shaped fully convolutional neural networks with multi-task learning. Compared to existing methods, this technique applies multi-task learning to predict the soma boundary to split touching somata, and adopts U-shaped architecture convolutional neural network which is effective for a limited dataset. The contour-aware multi-task learning framework is applied to the proposed method to predict the masks of neuronal somata and boundaries simultaneously. In addition, a spatial attention module is embedded into the multi-task model to improve neuronal soma segmentation results. The Nissl-stained dataset captured by the micro-optical sectioning tomography system is used to validate the proposed method. Following comparison to four existing segmentation models, the proposed method outperforms the others notably in both localization and segmentation. The novel method has potential for high-throughput neuronal soma segmentation in large-scale optical imaging data for neuron morphology quantitative analysis.

## Introduction

Neuron morphology is crucial for brain function research, such as electrophysiology simulation, connectome, and neuron type classification (Svoboda, 2011). On the one hand, the morphological features of neuronal somata are important for the quantitative analysis of neuron morphology when classifying neuron types (Peng et al., 2017). On the other hand, the distribution, ratio, number, and morphology of glial cells and neurons also contribute to the research on pharmacological treatment and pathology of the brain (Fitting et al., 2010; Attili et al., 2019). In addition, neuronal soma locations could be applied to enhance the results of neurite reconstruction (Zhang et al., 2018). Recently, with the rapid development in high-throughput optical micro imaging techniques (Gong et al., 2013; Wu et al., 2014), it is possible to acquire high-resolution large-scale neuron imaging datasets. However, current neuron reconstruction depends largely on manual labeling, which is error-prone and time-consuming (Acciai et al., 2016). As an alternative method, automated neuronal soma segmentation is highly efficient, and provides accurate results for neuronal somata morphology reconstruction (Meijering, 2010). The complexity of the optical imaging datasets causes many challenges for automated neuronal soma segmentation algorithms. First, the variable brightness in 3D optical imaging datasets, such as heterogeneous brightness between adjacent 2D imaging slices, and the weak signal region inside the neuronal soma, make it difficult to extract the regions that include the neuronal soma. Second, the neuronal soma has different shapes and sizes, and some neurons have an irregular-shaped soma. Most importantly, the images consistently show touching neuronal somata that are clustered in several local regions, with unclear boundaries between them. For these reasons, it is hard to localize or divide these neuronal into individual soma.

## Background

In recent decades, many studies have focused on cell segmentation. Early cell segmentation studies referenced image segmentation techniques. The most widely used technique is the intensity threshold method, which assumes a remarkable difference between the intensity of the foreground and background, and segments cells through a single threshold or a multilevel of thresholds. The intensity threshold can be calculated by image histograms (Otsu, 1973) and fuzzy sets (Pal et al., 2000). However, the intensity threshold method has difficulty in segmenting touching cells. While an improved threshold method using multi-level intensity to separate touching cells has been previously proposed (Keenan et al., 2000), since the touching cells have similar brightness and adjacent position (He et al., 2014), it remains challenging to perform accurate segmentation in this manner.

More recently, researchers have combined algorithms to avoid the shortcomings of individual methods under difficult conditions (Meijering, 2012). These methods contain seed-generating and segmenting methods where the initial seeds represent cell localization and work as initial markers for the generation of segmentation masks. The most widespread cell segmentation methods contain watershed algorithms, active contour models, and graph based methods. Watershed algorithm performs effectively for touching cells, but results in over-segmentation due to redundant seeds caused by low signal-noise-ratio and heterogeneous brightness in the images. To reduce redundant seeds, mark-controlled watershed algorithms are applied to cell segmentation (Yang et al., 2006). To achieve better the segmentation performance, a watershed algorithm using initial seeds generated by curvatures for cell segmentation in 3D confocal microscopy images was developed (Atta-Fosu et al., 2016). The level-set cell segmentation method, which segments by evolving the initial contour using an energy function, has been extended to 3D images and improved computation efficiency (Dufour et al., 2005). A subsequent study added the repulse item to their energy function to prevent touching cells from overlapping with one another (Yan et al., 2008). This method heavily depends on the initial contour, and while manual labeling is an accurate contour initialization method, it is very time-consuming for large-scale datasets. Al-Kofahi et al. (2010) proposed a two-stage method for cell segmentation in 2D images where a graph cut is applied in both stages to extract the foreground and optimize cell boundaries, respectively. While excellent for 2D images, many existing methods depend on brightness and gradient, which makes them unsuitable for 3D images containing variable brightness, gradient, and variable-shaped neuronal somata.

There are various studies on neuronal soma segmentation and detection for 3D images. Yan et al. (2013) proposed neuronal soma segmentation based on an improved rayburst sampling algorithm for a Golgi-stained dataset, but their method could not segment touching neuronal somata. He et al. (2014) combined the concave point detection and random walker methods for cell detection and cell segmentation, and found that the method works well in Nissl-stained dataset, especially for detecting touching cells. Cheng et al. (2016) proposed a touching neuronal soma localization method based on density peak clustering, which demonstrated a high detection accuracy. These combined methods predict accurate localization results for cells in 3D images. Nevertheless, there are few studies that focus on generating accurate segmentation masks of touching neuronal somata.

Accordingly, various deep learning-based methods have been proposed and found effective for instance segmentation or detection in medical image segmentation. Cireşan et al. (2013) proposed a cell detection method using convolutional neural networks (CNNs) which regard the cell detection task as pixel classification, and demonstrated excellent performance for Mitosis detection. Ronneberger et al. (2015) proposed a U-shaped fully convolutional neural network (FCN) for medical image segmentation which was found to be effective for limited datasets. To improve instance segmentation in medical image analysis, Chen et al. (2017) proposed deep contour-aware networks based on a multi-task learning framework. The method showed accurate segmentation of glands in colon histology images. The contour-aware networks can predict objects and contours simultaneously and learn discriminative features from complementary tasks to reduce the risk of overfitting. Importantly, the predicted contour is helpful for splitting touching objects. Recently, encoder-decoder FCNs (Khoshdeli et al., 2018) were used in nuclei segmentation and performed well for varying nuclear phenotypes. It should be noted that a weak supervised 3D neuronal network has been applied to neuronal soma segmentation (Dong et al., 2018).

In this study, we propose a method of 3D touching neuronal soma segmentation. The proposed method is based on a 3D FCN which combines a multi-task learning framework with U-shaped FCN. Additionally, a spatial attention module is embedded to learn representative features, and the total neuronal soma segmentation model contains 0.94 M parameters. This method is validated using Nissl-stained dataset captured by the micro-optical sectioning tomography (MOST) system.

## Methods

**Figure 1:**
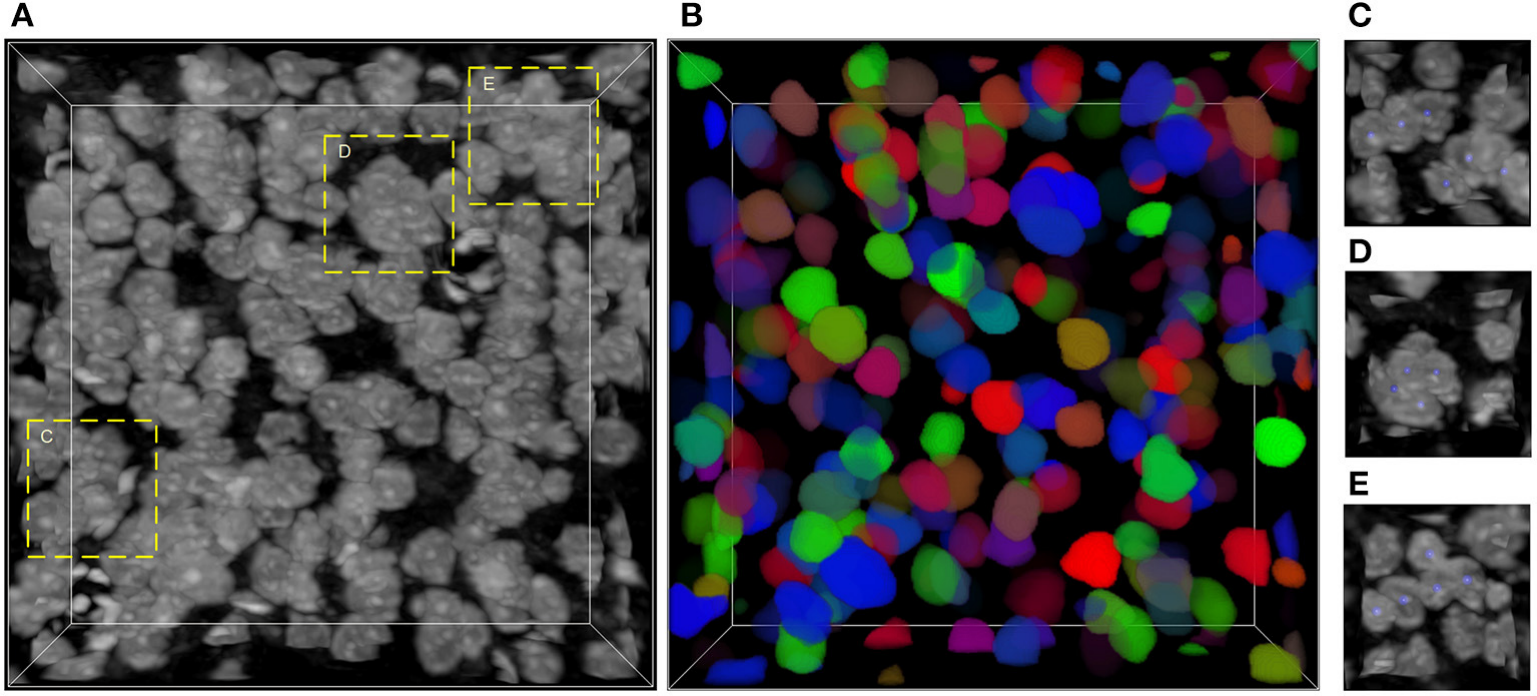
Nissl-stained dataset. **(A)** Raw volumetric data. **(B)** Manual labels; each neuronal soma is labeled by unique random color. **(C–E)** Regions contain touching neuronal somata, labeled by dash bounding boxes in **(A)**; the neuronal soma centers are labeled using blue balls. The intensity of raw data is adapted for visualization. The shape of this volumetric data is 286 × 286 × 86 voxels, and the voxel size is 0.35 × 0.35 × 0.35 μm.

The main challenge of neuronal soma segmentation is the handling of touching neuronal somata. Figure 1 depicts the Nissl-stained dataset used in this study. As shown in Figures 1C–E, there are many touching neuronal somata distributed throughout the dataset, and the boundaries between touching somata are blurred because of similar brightness. This makes it difficult to divide touching neuronal soma clusters into individuals ones. In this study, we proposed 3D mutli-task U-shaped fully convolutional neural network. Specially, the neuronal soma segmentation is decomposed into two complementary tasks, namely predictions of neuronal soma segmentation and boundary, that applied to split the touching neural somata. The backbone of the proposed fully convolutional neural networks is designed based on U-shaped architecture which is effective for training in limited data.

### Process Overview

**Figure 2:**
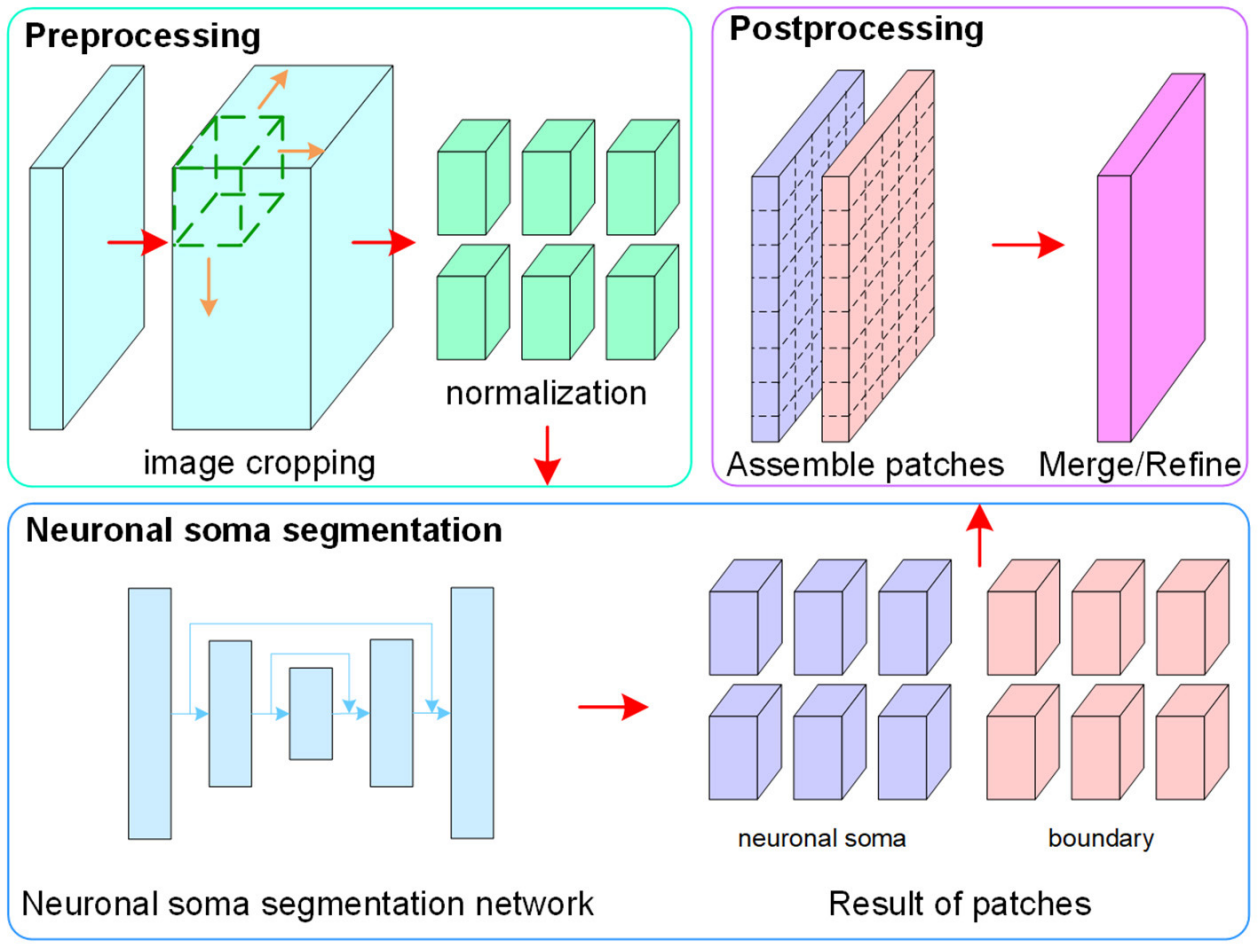
Flowchart of the proposed method.

The flowchart of the proposed method, which consists of image pre-processing, neuronal soma segmentation, and post-processing, is shown in Figure 2. In the pre-processing step, raw data is normalized and cropped into small patches to reduce the memory footprint during the training and testing stages. In the second step, the FCN is used to predict regions containing the neuronal soma and boundary for each neuron. Finally, the post-processing step is used to assemble and merge the results of the patches.

### Pre-processing

**Figure 3:**
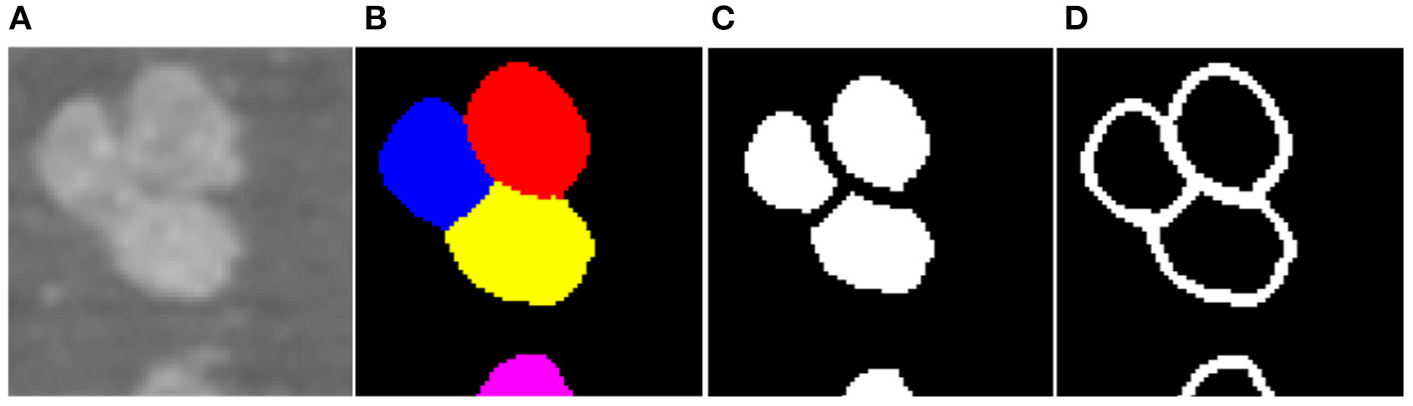
Sample labels. **(A)** Raw image slice. **(B)** Manually labeled neuronal somata, individual soma is labeled by unique random color. **(C)** Unconnected neuronal somata label. **(D)** Boundary label. The neuronal soma region and boundary region have a complementary spatial arrangement.

To facilitate the training of the CNN, z-score normalization is performed on the raw data. The mean value and standard deviation are evaluated from all voxels collected in the training set. In addition, the original random color labels of the neuronal somata are transformed into binary labels including the neuronal soma and boundary. Figure 3 shows the sample labeling. As illustrated in Figure 3C, the neuronal soma regions are unconnected for each neuronal soma. Figure 3D shows the boundaries extracted from the original random color labeled somata (Figure 3B). To alleviate sample imbalance in the boundary label, a binary dilation operator (with ball structuring element with radius of 1 voxel) is used to increase the size of the boundary regions.

The original size of the volumetric data is 285 × 285 × 86 voxels, which is too big for model training using limited GPU memory. Therefore, the raw data is cropped into small patches with a size of 80×80×80 voxels. The mean neuronal soma radius in this dataset is 11 voxels. As this is significantly smaller than the original image size, using smaller patches should not influence the performance. To make full use of the samples, a slide window with a stride of 48 voxels is used to generate the patches. After cropping, there are overlapping regions that are 32 voxels wide left in adjacent patches, and the neuronal somata broken by the patch borders are expected to present completely in at least one patch (Yan et al., 2013). This cropping operation runs in the same way for both the training and testing stages.

### Neuronal Soma Segmentation

**Figure 4:**
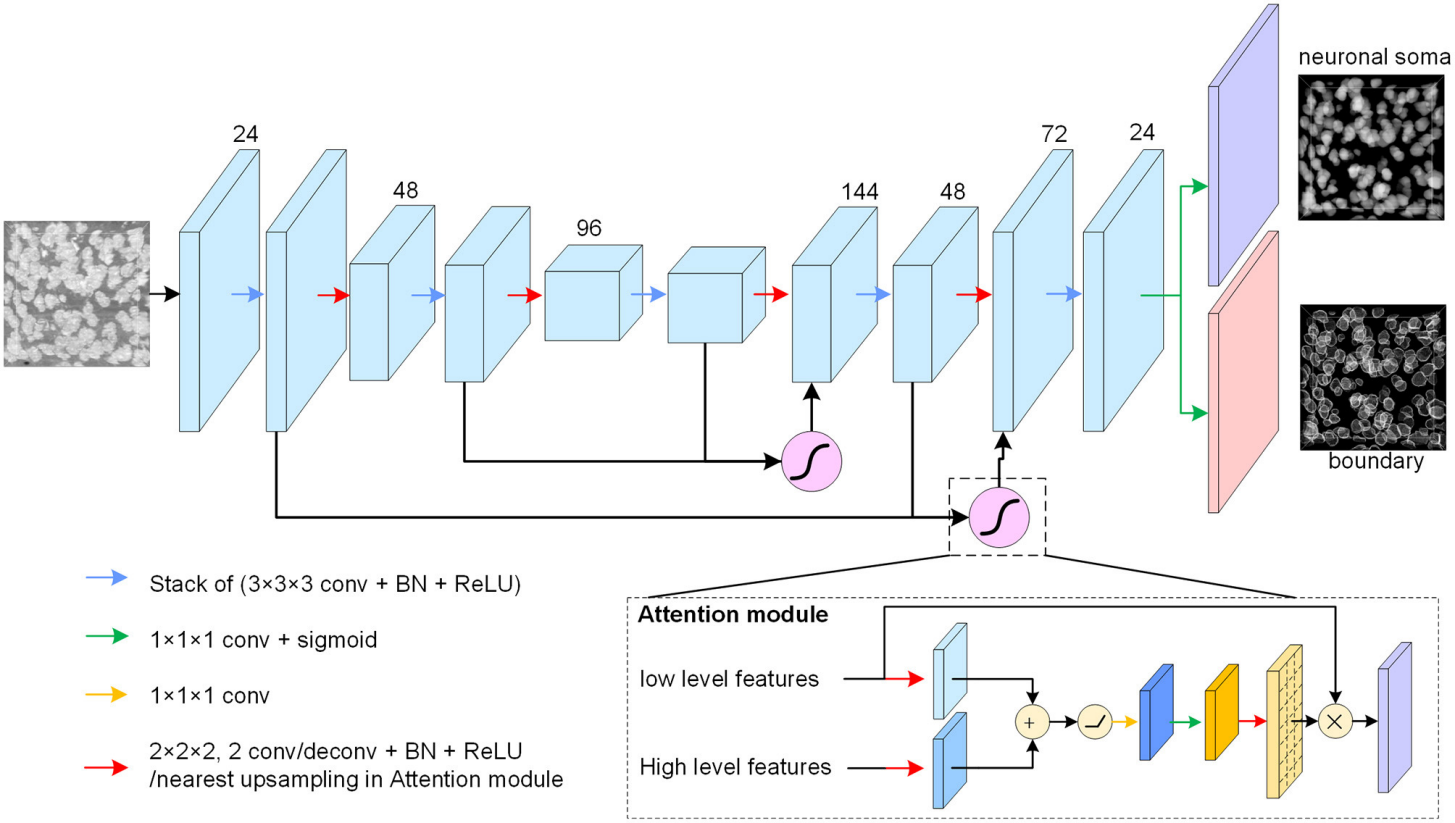
Neuronal soma segmentation model. The blocks represent the feature maps. The violet and red blocks denote the two output branches of boundary and neuronal soma, and the purple module is the spatial attention module. The kernel size of the 3D convolutional layers is 3 × 3 × 3, and that of the prediction layer is 1 × 1 × 1. The root feature map number is 24. The black arrow indicates data flow, and the green and yellow arrows represent the 1 × 1 × 1 convolution layer with or without sigmoid activation, respectively. The red arrow represents the down-sampling or up-sampling layers in the CNN. The down-sampling layer is a convolution layer with a stride of 2, and the up-sampling layer is a deconvolution layer with a stride of 2. In particular, the up-sampling layer in the attention module is the nearest interpolation.

The neuronal soma segmentation network is the main part of the segmentation pipeline shown in Figure 2. The state-of-the-art U-shaped FCN (Ronneberger et al., 2015) applied in the proposed model predicts the boundary and neuronal soma locations simultaneously, as illustrated in Figure 4.

The proposed FCN architecture model includes an encoder and a decoder for dense prediction. 3D convolution and deconvolution layers are applied in the model to explore the 3D spatial information from the raw images. The encoder is used to extract features from raw images using stacks of convolution layers, and the down-sampling layer is applied to reduce feature map size and enlarge the receptive field. In this study, convolutional layers with strides of two substitutes for max-pooling layers are used in the U-net. The number of feature maps is doubled after reducing the resolution.

The decoder recovers the feature map resolution gradually. It contains a trainable deconvolution layer which up-samples feature maps by a factor of two, following stacks of convolutional layers to reduce the number of feature maps gradually. Specially, the neuronal somata is small comparing with input image shape (the mean radius of neuronal soma is about 5 μm (Yan et al., 2013), is about 15 voxels in Nissl-stained dataset). The volume of neuronal soma could be few voxels after the processed by some down-sampling layers. For this reason, fewer down-sampling layers are applied in the proposed model. As illustrated in Figure 4, the architecture of model is symmetric and comprises two down-sampling layers and two up-sampling layers.

There are skip connections between the encoder and decoder which combine feature maps with different resolutions. The output branch for the neuronal soma and boundary share the feature maps from the U-shaped FCN and predict the results using a convolution layer with a kernel size of 1×1×1 following sigmoid activation. All convolution layers, except the spatial attention module and output layers, are followed by batch normalization layers and Rectified Linear Unit (ReLU) activation to accelerate model convergence. In addition, the feature maps from the deeper layers are semantically strong but have few spatial details, whereas the feature maps captured by the shallow layers contain rich detail. U-shaped architecture, therefore, tries to recover the missing details by combining feature maps from the adjacent levels through skip connections (Ronneberger et al., 2015). As there are some heterogeneous brightness regions in the raw data which could be ambiguous for prediction, a spatial attention module (Oktay et al., 2018) is applied to extract related regions from low level feature maps before merging feature maps of different resolutions.

As shown in Figure 4, the attention module receives the feature maps from adjacent levels as inputs and learns to suppress irrelevant regions in the low level feature maps. The input feature maps are merged by an addition operation, and successive linear transform layers (convolutional layers with kernel size of 1 × 1 × 1) are used to calculate the attention coefficient. In this study, the linear transform layers do not reduce the number of feature maps, and the nearest interpolation is used to up-sample the attention coefficient instead of the tri-bilinear interpolation used by Oktay et al. (2018). Thereafter, the low-level feature maps are weighted by attention coefficient and combined with the high level feature maps.

During the training stage, the loss function guiding the model parameter update is the sum of the cross-entropy *L*_*ce*_ and soft-Dice loss *L*_*Dice*_, where the soft-dice loss is applied to deal with the imbalance problem in the boundary branch (Milletari et al., 2016). The above-mentioned terms are defined in the following equations:

(1) $$\begin{array}{l} L_{ce}\;=\;-\frac{1}{M}\underset{i\in N}{\sum }\left(y_{i}log\left(p_{i}\left(x_{i},w\right)\right)+\left(1-y_{i}\right)log\left(1-p_{i}\left(x_{i},w\right)\right)\right) \end{array}$$

(2) $$\begin{array}{c} L_{Dice}=\frac{2\;\times \sum_{i\in N}y_{i}\;\times \;p_{i}(x_{i},w)}{\sum_{i\in N}p_{i}(x_{i},w)+\sum_{i\in N}y_{i}} \end{array}$$

(3) $$\begin{array}{l} L_{branch}=L_{ce}+L_{Dice} \end{array}$$

where *i* indicates the voxel position in the image space *N, M* is the number of the total voxels, *y*_*i*_ and *x*_*i*_ are the respective label and sample for one voxel, and *p*_*i*_(*x*_*i*_, *w*) is the predicted result of the proposed model *w*. This loss is used for the segmentation of the both the boundary and neuronal soma, and the total loss is defined using Equation (4).

(4) $$\begin{array}{l} L_{total}=L_{bou}+L_{soma} \end{array}$$

*L*_*soma*_ and *L*_*bou*_ represent the losses of the branches of the neuronal soma and boundary, respectively.

### Post-processing

In the testing stage, the neuronal soma segmentation network predicts the boundary and object regions for each patch from the same image. The post-preprocessing step assembles the predicted patches into the original shape and refines the segmentation result.

Patches from the same image are assembled according to their original locations in the raw image. In the cropping step, overlapping regions are left to make full use of the samples. However, as regions near the patch borders do not provide enough contextual information for accurate prediction, only the center regions in each patch are extracted to be assembled as the final result for fast predicting. Second, the probability maps are clipped at a threshold of 0.5 to generate binary masks, following which the boundary region is subtracted from the object region to split the connected touching somata. Finally, marker-controlled watershed is used to fill the gap left by the subtracted boundary region. Besides, the center points of each predicted masks for individual neuronal soma are extracted as localization results.

### Implementation

In the pre- and post-processing steps, a ball structuring element with a radius of one is used for the morphology operations of the dilation operator (in label generating) and of the opening operator (in result refining), respectively. The sliding window size is set to 80 × 80 × 80 with a 32 voxels-wide overlapping region for adjacent patches.

During model training, on-the-fly random flipping and random brightness are used to augment the samples. The Adam optimizer (Kingma and Ba, 2014) with an initial learning rate of 0.001 is used to train the model. The early-stopping strategy is used to select the best model and each epoch contains 100 iterations. Additionally, touching boundary recalls are also considered as auxiliary measures for model selection. The mini-batch size is set to four in this study.

The implementation and dataset with annotation of proposed method will be available soon in https://github.com/keepersecond/neuronal-soma-segmentation.

## Results

In this study, we compare the proposed method with several 3D neuronal soma segmentation methods in Nissl-stained dataset, including the concave points clustering (CPC) random walker algorithm (He et al., 2014), the distance transform-based rayburst sampling algorithm (Hu et al., 2017), and 3D FCNs (3D UNet, Vnet) (Çiçek et al., 2016; Milletari et al., 2016). To validate the deep learning results similarly, all the methods are evaluated using a 3-fold cross-validation, where the dataset is split into three groups (seven images, seven images, six images). The pre- and post-processing steps of this study are also applied in other 3D CNNs, and the weighted cross-entropy loss function (Ronneberger et al., 2015) proposed for cell segmentation is also used in other 3D CNNs. The parameters of the CPC random walker and rayburst sampling algorithms are set as defaults.

To validate performance in dataset captured by different system and labeled by different staining method, we test the proposed method in green fluorescence protein (GFP) labeled dataset captured by fluorescence microscope optical section tomography (fMOST) system (Gong et al., 2013).

All the methods were validated on an Intel Xeon® E5-2630 2.4 GHz workstation with 64 GB RAM, and a NVIDIA TITAN Xp graphics card.

### Dataset

The test datasets of the current study contain two parts. The first comes from a C57B/L mouse, and was captured by Nissl-staining method and the MOST system (Wu et al., 2014). The original voxel size is 0.35 × 0.4 × 1 μm. The raw data selected from the cortex was resized by cubic interpolation to achieve an isotropic resolution of 0.35 × 0.35 × 0.35 μm and processed by removing noise and correcting intensity. To validate the proposed model, 20 image stacks with dimensions of 285×285×86 voxels are extracted. The total dataset contains ~4,000 manually labeled neuronal somata. Unclear structures in the raw data are ignored.

Another test dataset comes from Thy1-GFP-M mouse, and was captured by fluorescence micro-optical sectioning tomography (fMOST) system (Gong et al., 2013). The original voxel size is 0.5 × 0.5 × 2 μm. The raw data was selected from cortex and resized to achieve and isotropic resolution of 0.5 × 0.5 × 2 μm, and 34 image stacks with dimensionals of 200 × 200 × 200 voxels are extracted from an original image data (921 × 1,435 × 200 voxels). This dataset contains about 300 manually neuronal somata.

### Evaluation of Soma Segmentation

The segmentation result is validated in terms of soma localization and segmentation. The localization performance is evaluated by recall, precision, and F1 score. One neuronal soma is correctly detected if it matches one ground truth. The mass centers of the manually labeled somata and segmentation results are regarded as ground truth and prediction results, respectively. If the distance between the ground truth and result is less than the mean soma radius Rc, the pair is matched. Rc is set to 11 voxels in Nissl stained dataset and 13 voxels in GFP dataset. The recall, precision, and F1 score are defined using Equations (5–7), respectively:

(5) $$\begin{array}{l} precision=\frac{N_{tp}}{N_{fp}+N_{tp}} \end{array}$$

(6) $$\begin{array}{l} recall=\frac{N_{tp}}{N_{fn}+N_{tp}} \end{array}$$

(7) $$\begin{array}{l} F1=\frac{2\;\times \;precision\;\times \;recall}{precision+recall} \end{array}$$

where *N*_*tp*_ represents the number of true detected somata, *N*_*fp*_ represents the number of missed neuronal somata, and *N*_*fn*_ represents the number of falsely detected neuronal somata.

The segmentation performance is evaluated by Dice coefficient, which describes the overlapping ratio of ground truth to segmentation result. The ground truth and result are set as masks of individual soma in the manual label and predict the result correspondingly. The Dice coefficient is defined using Equation (8):

(8) $$\begin{array}{l} Dice=\frac{2\;\times \;Seg\cap Gt}{Seg\cup Gt} \end{array}$$

where *Seg* and *Gt* are the masks of the neuronal soma in the ground truth and segmentation result, respectively. Only the correctly detected neuronal somata are evaluated by Dice coefficient (Yan et al., 2013).

**Figure 5:**
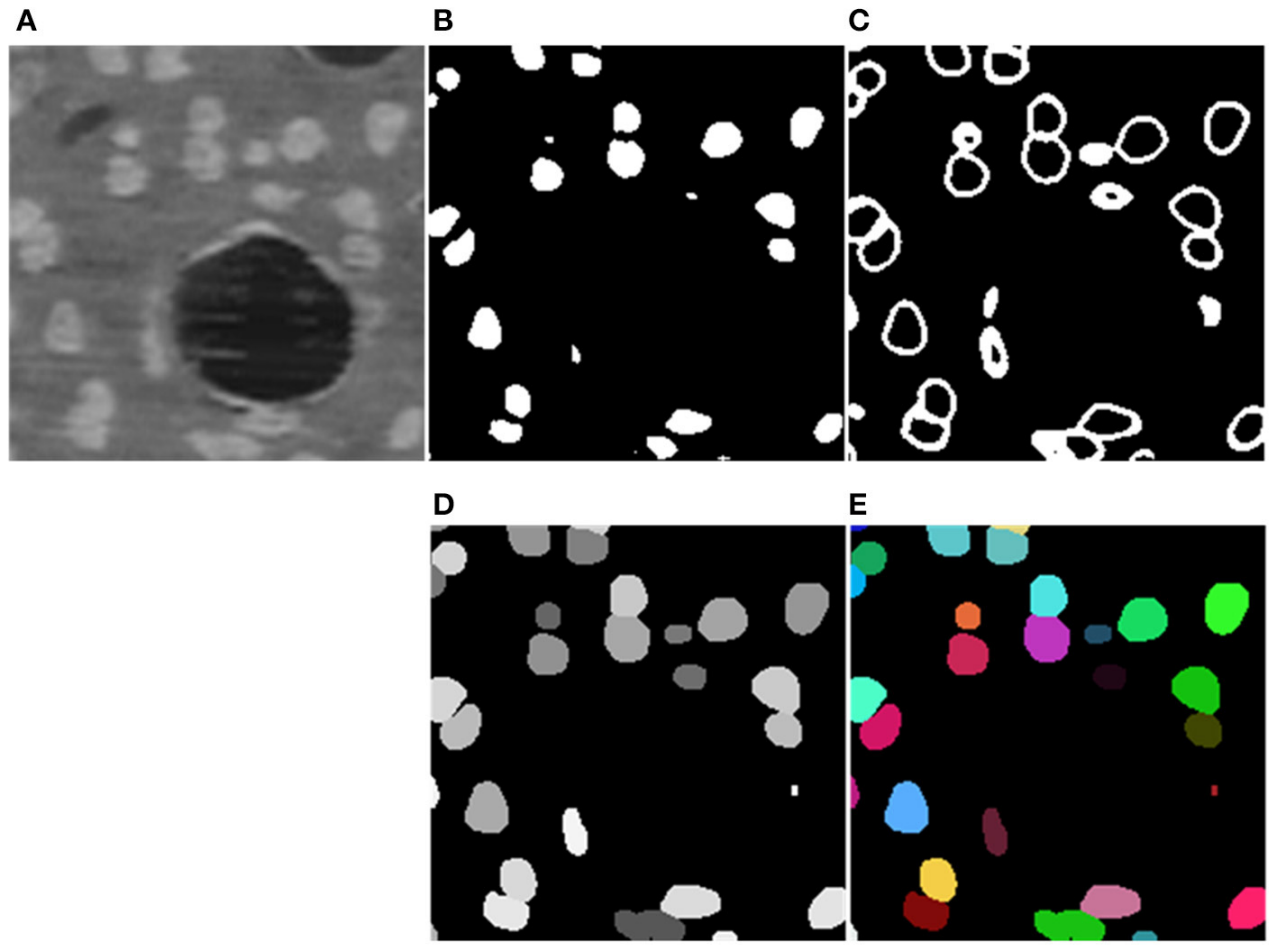
Segmentation Steps. **(A)** Raw data. **(B,C)** Predicted boundary and object for each neuronal soma. **(D)** Refined segmentation results where individual neuronal somata are labeled by unique gray levels. **(E)** Neuronal soma labeled by unique random color.

The segmentation results of the proposed method are shown in Figure 5. The model generates results for the boundary and neuronal soma simultaneously and yields complete boundary predictions for individual touching neuronal somata. Table 1 compares the performance of the neuronal soma localization among different methods.

**Table 1:** Comparison of performance of neuronal soma localization results using different methods.

**Method**		**Recall**	**Precision**	**F1**
CPC-walker	Fold1	0.91	0.64	0.74
	Fold2	0.92	0.61	0.73
	Fold3	0.92	0.67	0.77
	**Average**	**0.92**	**0.64**	**0.75**
Rayburst	Fold1	0.76	0.74	0.74
	Fold2	0.77	0.67	0.70
	Fold3	0.78	0.79	0.78
	**Average**	**0.77**	**0.73**	**0.74**
3D UNet	Fold1	0.88	0.73	0.79
	Fold2	0.94	0.85	0.89
	Fold3	0.90	0.91	0.90
	**Average**	**0.91**	**0.83**	**0.86**
VNet	Fold1	0.90	0.79	0.84
	Fold2	0.94	0.88	0.91
	Fold3	0.87	0.88	0.87
	**Average**	**0.90**	**0.85**	**0.87**
Proposed	Fold1	0.91	0.93	0.92
	Fold2	0.89	0.94	0.92
	Fold3	0.90	0.96	0.93
	**Average**	**0.90**	**0.94**	**0.92**

As shown in Table 1, at 0.92, the CPC walker algorithm has the highest recall ratio. While this method detects more neuronal somata than other methods, it has a notable false localization rate, which lowers its precision. The rayburst sampling algorithm demonstrates similar precision and recall, but at 0.74, has the lowest F1 score. Being based on 3D FCNs, both the 3D UNet and VNet methods as well as the proposed method offer a better trade-off between recall and precision. Compared with the 3D UNet and VNet models, our proposed method demonstrates higher precision and a comparable recall ratio, achieving the best F1 score of 0.92.

**Figure 6:**
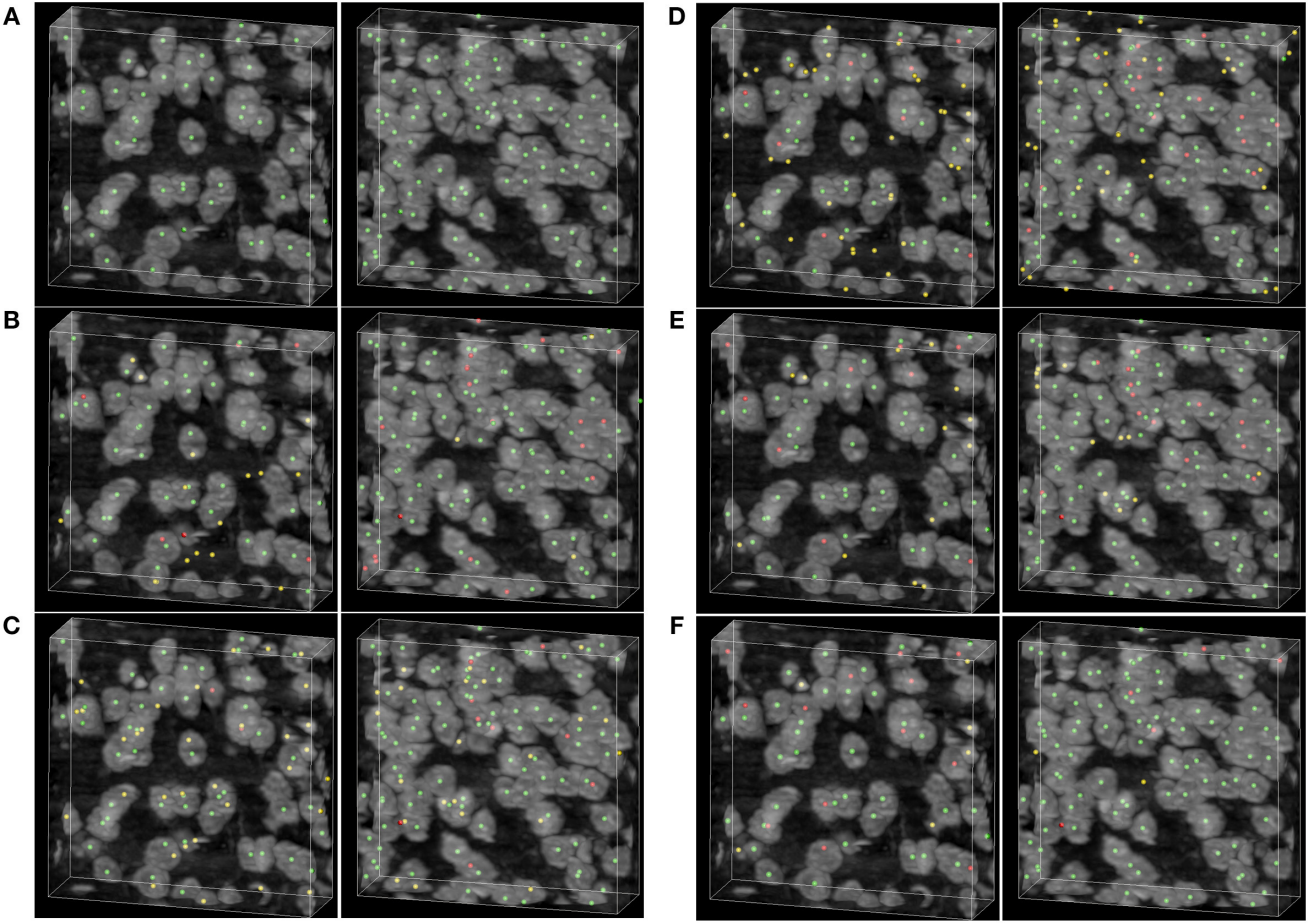
Localization results. **(A)** Ground truth; the neuronal soma location is demonstrated by neuronal soma centers labeled using green balls. **(B)** Distance transform based rayburst sampling algorithm, **(C)** CPC random walker **(D,E)** 3D UNet and VNet, respectively. **(F)** Proposed method. The falsely detected somata are labeled using yellow balls and the missing neuronal somata are labeled using red balls. The shape of the volumetric data is 200×200×60 voxels, and the voxel size is 0.35 × 0.35 × 0.35 μm.

To demonstrate the detecting result of the methods, two image stacks with dimensions of 200×200×60 voxels are extracted from the raw dataset shown in Figure 6. As can be seen in Figures 6B–F, the rayburst sampling algorithm misses many neuronal somata, and the main error of the CPC random walker and 3D UNet methods comes from false localization. The VNet and the proposed method demonstrate better performance than the other methods, and there are less falsely detected neuronal somata in the result of proposed method than in the VNet method. Table 2 shows the results of the soma segmentation.

**Table 2:** Evaluation of soma segmentation by comparing Dice coefficients.

**Method**	**Fold1**	**Fold2**	**Fold3**	**Average**
CPC-walker	0.7742	0.7711	0.7728	0.7727
Rayburst	0.7233	0.7276	0.7320	0.7276
3D UNet	0.7486	0.8269	0.8385	0.8047
VNet	0.8404	0.8372	0.8192	0.8323
Proposed	0.8024	0.8533	0.8750	0.8436

The under- and over-segmentation is used to analyze the segmentation performance. Over-segmentation indicates that one neuronal soma has been split into pieces, and results in the false localization of neuronal somata and a low Dice coefficient. On the contrary, under-segmentation means that several neuronal somata have been segmented as one neuronal soma.

**Figure 7:**
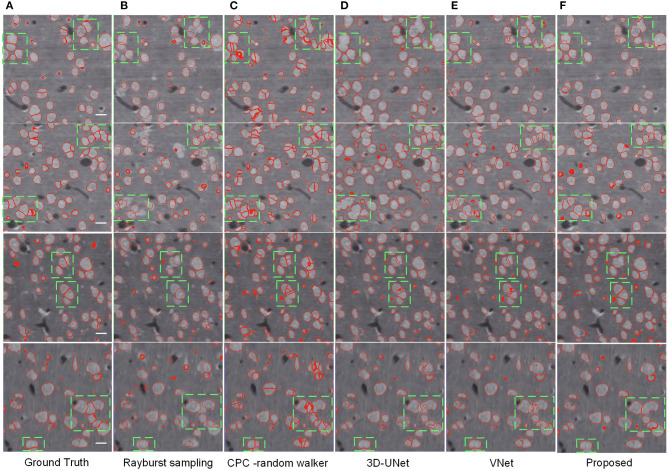
Segmentation results of different methods. **(A)** Ground truth; boundary of random color label is used to represents segmentation result, the slices are extracted from three different volumes. The horizontal bar indicates a distance of 10 μm. **(B)** Distance transform based rayburst algorithm. **(C)** CPC random walker. **(D,E)** 3D UNet and VNet with spatial weighted cross-entropy loss, respectively. **(F)** Proposed method. Several typical regions, including touching neuronal somata are extracted by green bounding boxes for comparing the methods.

As shown in Table 2, the CPC random walker algorithm has an average Dice coefficient of 0.7727, and that of the rayburst sampling algorithm is 0.7276. Outdoing the two methods, the 3D FCN models demonstrate average Dice coefficients which are higher than 0.8. At 0.8436, the proposed method achieves the best Dice coefficient, which indicates that it can generate accurate contours for neuronal somata. Figure 7 shows the segmentation results of the evaluated methods.

As can be seen in Figure 7B, the rayburst sampling algorithm generates ellipsoid segmentation masks for the neuronal somata. The main error is under-segmentation, and the ellipsoid model is not suitable for irregular-shaped neuronal soma or touching neuronal somata. The CPC random walker is able to segment nearly all the neuronal somata but makes many over-segmentations for both the isolated and touching neuronal somata (as shown in Figure 7C). This suggests that the relatively low average Dice coefficient of 0.7727 shown in Table 2 may have been influenced by the prevalence of wrongly segmented neuronal somata. The 3D FCN based models demonstrate better segmentation results, and the main error is under-segmentation of the touching neuronal somata. The VNet seems to predict less false located regions than the 3D UNet. Notably, compared with the 3D UNet and VNet, the proposed method predicts better contours for multiple touching neuronal somata than the VNet (as illustrated downmost in Figures 7E,F).

**Figure 8:**
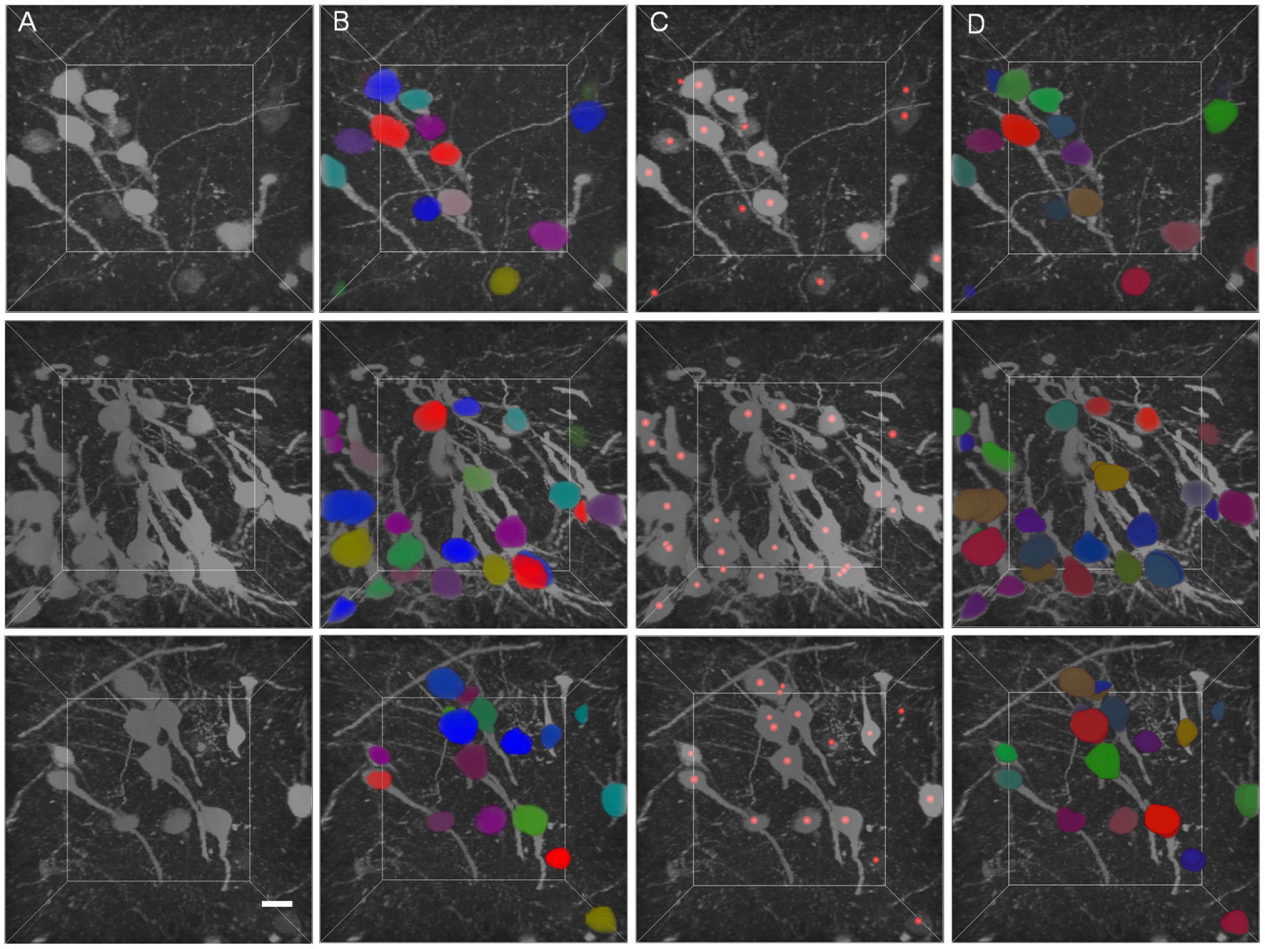
Validation of proposed method in GFP dataset. **(A)** Raw volumetric data from GFP dataset. **(B)** Manual labels. **(C)** localization results. **(D)** Segmentation results. Each neuronal soma in **(B)** and **(D)** is labeled by unique random color. The neuronal soma localization results are represented by red balls. The intensity of raw data is adapted for visualization. The shape of each volumetric data is 200 × 200 × 200 voxels, and the voxel size is 0.5 × 0.5 × 0.5 μm. The horizontal bar indicates 10 μm.

The segmentation results of GFP dataset is shown Table 3, the proposed method achieve a comparable performance in soma localization and segmentation, the F1 score of localization is 0.89, average dice of segmentation is about 0.87. As illustrated in Figure 8, the challenge of soma segmentation in GFP dataset is neurites with high intensity around neuronal soma, which could result in false positive in localization, the proposed method could suppress the neurites and segment neuronal soma accurately (as shown in Figure 8D).

**Table 3:** Evaluation of soma segmentation in GFP dataset captured by fMOST system.

	**Fold1**	**Fold2**	**Fold3**	**Average**
Recall	0.9164	0.9538	0.9335	0.9345
Precision	0.8854	0.8475	0.9089	0.8806
F1	0.8915	0.8874	0.9162	0.8983
Dice	0.8519	0.8693	0.8851	0.8688

## Discussion

This study proposes a neuronal soma segmentation method based on a U-shaped FCN. The method is trained on a small dataset and demonstrates obvious performance. We compared the proposed method to the CPC random walker algorithm, rayburst sampling algorithm, and two advanced 3D FCNs (3D UNet and VNet) for medical image segmentation. The proposed method outperforms the others in localization and segmentation.

Over-segmentation is significant in the CPC random walker for isolated neuronal soma, and is presumably caused by heterogeneous brightness on the soma surface (He et al., 2014). There is also over-segmentation observed in touching neuronal somata. This could be caused by the localization of false concave points on the surfaces of irregular-shaped touching neural somata. The rayburst sampling algorithm displayed over-segmentation and under-segmentation in the Nissl-stained dataset. It is thought that the ellipsoid model could not accurately describe the irregular-shaped neuronal somata, and may have split the elongated somata into multiple ones or missed the plat-shaped somata (Hu et al., 2017). These methods assume that the neuronal soma is ball-like or ellipsoid-like, which may not suit irregular-shaped neuronal somata. The FCNs show better performance in both localization and segmentation, which is most likely due to their encoder-decoder architecture (Khoshdeli et al., 2018). Compared with the CPC random walker and rayburst sampling algorithms, the FCNs were able to learn effective feature representation from raw images and make accurate predictions.

In this study, the neuronal soma segmentation was separated into two complementary tasks, namely predictions of boundary and neuronal soma position. This multi-task model has the advantage of learning discriminating features for model prediction and reducing overfitting (Chen et al., 2017). The proposed model, with only 0.94 M parameters, shows better performance than the 3D UNet (19 M trainable parameters) and VNet (65 M trainable parameters) using weighted cross-entropy loss. Besides, this study uses an attention gate module to improve the performance of neuronal soma segmentation through surpassing unrelated regions. A slight improvement of 1.5% is observed in the F1 score in soma localization.

Besides, the proposed method has been validated in dataset labeled by green fluorescence protein (Gong et al., 2013). The appearance of this dataset is different from the Nissl stained dataset. There are many neurites with high intensity around the neuronal somata, which makes it difficult to extract neuronal soma regions. The results show that the proposed method could segment neuronal soma accurately in this dataset (as shown in Table 3), suppress interference from neurites (as shown in Figure 8). The proposed method has potential for neuronal soma segmentation in dataset captured by other staining method.

**Figure 9:**
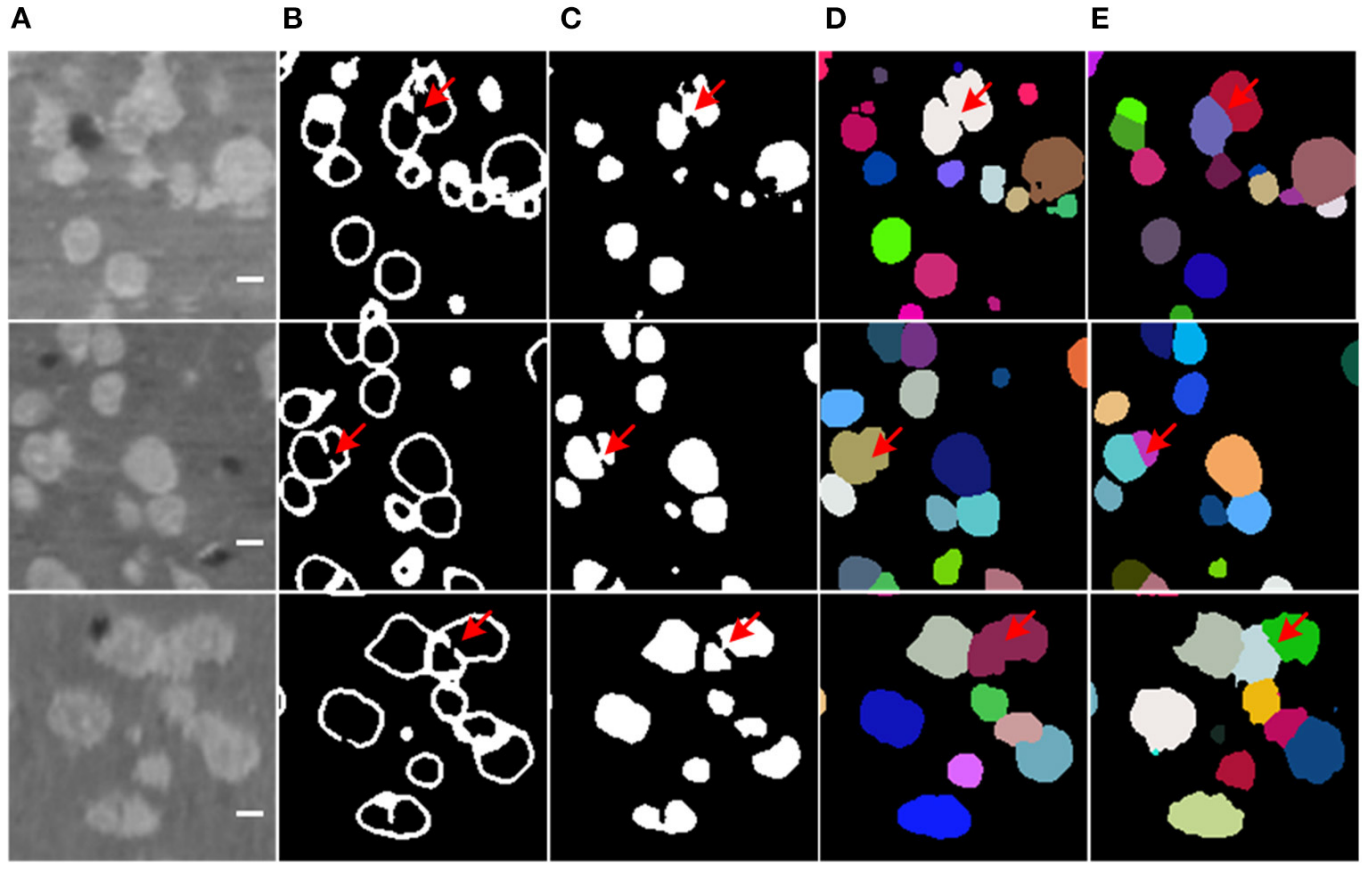
Missing boundary in results. **(A)** Slices from raw volumetric data. **(B)** Predicted mask of boundary. **(C)** Predicted mask of neuronal soma. **(D)** Segmentation result labeled by random unique color. **(E)** Ground truth. The shape of slices is 150 × 150 voxels, the bar indicates 5 μm, and the red arrows label the local missing boundaries.

Additionally, the boundary prediction helps to split the touching neuronal somata. In many cases, the proposed model successfully predicts touching boundaries, even for multiple touching neuronal somata (as shown in Figure 7). Nevertheless, the main error in our proposed method comes from under-segmentation caused by local boundaries missing in the prediction result (as shown in Figure 9), though Dice loss has been used to deal with the sample imbalance in the boundary branch. The possible reason could be heavy sample imbalance and the presence of hard samples in the boundary region. Clearly, the touching neuronal boundary is the key to splitting the clustered somata; however, seeing as the ratio of touching boundaries to total boundary voxels was <10%, it could not be processed by the soft-Dice loss used in the boundary branch. Moreover, it is believed that the touching boundary could be hard to learn, or the voxels in the touching boundary could have a different neighborhood to those of the boundary between the background and foreground (as shown in Figure 3). For this reason, the touching boundary could be mistaken for a region inside the neuronal soma with similar brightness. The focal loss (Lin et al., 2017) proposed for dealing with hard examples and a more powerful encoder (He et al., 2016; Huang et al., 2017) could be possible solutions to this problem. Furthermore, the object detection framework could avoid the heavy sample imbalance in the voxel-wise boundary and provide a better localization for neuronal somata.

Moreover, the proposed method regards the neuronal soma segmentation as voxel-wise prediction task in an end-to-end way. Though the neuronal soma size is a basis of network design, information about neuronal soma morphology should not be ignored. For example, the neuronal somata are always blob-like, which is different from neurites and vessel. It is an important cue to distinguish the neuronal soma from other structure or split touching neuronal soma with similar intensity. Specially, multiscale orientable filters have been applied in the neuronal soma segmentation to identity the blob-like structure, this method can distinguish the blob-like soma from contiguous neurites efficiently (Kayasandik and Labate, 2016). Besides, gas of circles (GOC) active contour model is applied to fluorescently stained cell segmentation, this model is initialized by a circular marker and segment overlapping cell accurately (Molnar et al., 2016). These works have proven that the soma morphology prior knowledge can process the unrelated structure and clustered cells. In this study, experiment shows that the proposed method could predict unclear touching boundary accurately (as illustrated in Figure 8). Integrating the morphology information into Loss functions or post-preprocessing step of FCN could be efficient way to improve the robustness of algorithm to resist the disturbance of unrelated structure and split touching soma with unclear touching boundary.

## Conclusion

In this study, a deep learning-based method is proposed for 3D neuronal soma segmentation. The main part of the method is the neuronal soma segmentation network, which is a multi-task learning U-shaped fully convolutional neural network into which a spatial attention module is embedded to improve the performance. This model can predict the respective masks of neuronal soma and boundary. The total model contains only 0.94 M trainable parameters, reducing the risk of over-fitting. The proposed method can segment touching neuronal soma and irregular-shaped soma efficiently and generate accurate contours for individual neuronal soma with simple post-processing based on watershed transform.

The methods are validated in Nissl stained dataset captured using the MOST system. The proposed method outperformed four existing neuronal soma segmentation methods by achieving an F1 score of 0.92 and an average Dice coefficient of 0.84, respectively. Compared with 3D FCNs which have more trainable parameters (19 M parameters in 3D UNet, 65 M parameters in VNet), the proposed method generated fewer false detected targets and achieved comparable recall in neuronal soma localization. In addition, the proposed U-shaped neuronal soma segmentation network can be trained with limited training data.

The proposed method has potential for high-throughput neuronal soma segmentation in large-scale 3D optical imaging data and provides accurate neuronal soma contour for neuron morphology analysis in brain function research.

## Data Availability Statement

The data can be downloaded from https://github.com/keepersecond/neuronal-soma-segmentation/releases/download/0.1/nissl-staining-images.zip.

## Ethics Statement

The animal study was reviewed and approved by Institutional Animal Ethics Committee of Huazhong University of Science and Technology.

## Author Contributions

QL and SC conceived the project. TH designed the method and wrote the article. XX organized and processed the datasets. All authors contributed to the article and approved the submitted version.

## Conflict of Interest

The authors declare that the research was conducted in the absence of any commercial or financial relationships that could be construed as a potential conflict of interest.
